# Patients´ satisfaction concerning direct anterior dental restoration

**DOI:** 10.1590/0103-6440202305260

**Published:** 2023-07-17

**Authors:** Bruna Neves de Freitas, Paulo Oliveira da Silva, Karen Pintado-Palomino, Cecília Vilela Vasconcelos Barros de Almeida, Aline Evangelista Souza-Gabriel, Silmara Aparecida Milori Corona, Saulo Geraldeli, Brigitte Grosgogeat, Jean-François Roulet, Camila Tirapelli

**Affiliations:** 1 Department of Dental Materials and Prosthodontics, School of Dentistry of Ribeirão Preto, University of São Paulo. Ribeirão Preto, SP, Brazil.; 2 College of Dentistry, University National San Luis Gonzaga. Ica, Peru.; 3 Department of Prosthodontics and Bucofacial Surgery, Federal University of Pernambuco. Recife, PE, Brazil.; 4 Department of Restorative Dentistry, School of Dentistry of Ribeirao Preto, University of São Paulo. Ribeirão Preto, SP, Brazil.; 5 East Carolina University, School of Dental Medicine, Department of General Dentistry Greenville. Greenville, NC, United States of America.; 6 Faculté d’Odontologie, Laboratoire des Multimateriaux et Interfaces, UMR CNRS 5615, France.; 7 Pôle d’Odontologie, Hospices Civils de Lyon, Lyon, France.

**Keywords:** patient satisfaction, FDI criteria, clinical decision-making, composite resins, clinical studyx

## Abstract

The objective of this study was to observe patients’ satisfaction with their in-service direct anterior dental restorations and to compare it with clinical evaluation using FDI (Federation Dental International) criteria. Patients scored their own anterior dental restorations regarding satisfaction (satisfactory /dissatisfactory). If dissatisfaction was mentioned, then, they would be interviewed about the complaint. In the same session, the dental restorations were clinically evaluated by two dentists using FDI criteria (1-5 score) concerning esthetic, functional, and biological domains. Descriptive statistics were used for frequencies of scores attributed by patients and clinicians. In order to compare patients’ to clinicians’ frequencies, the Chi-square test was applied (p ≤ 0.05). A total of 106 restorations were evaluated by patients and clinicians. Patients reported 52.8% of restorations satisfactory and 47.8% dissatisfactory. Overall, clinicians reported the same restorations as 82,3% satisfactory and 17,6% dissatisfactory. Patients’ most frequent complaints referred to color, followed by anatomical form, fracture of material and retention, and approximal anatomical form. Comparing patients’ satisfaction and dissatisfaction rates to clinicians’ evaluation per criteria, there was no difference regarding esthetics. The frequency of dissatisfactory restorations by clinicians was significantly lower when functional and biological properties were compared with patients’ opinions. Direct anterior dental restorations were more frequently reported as satisfactory by patients and clinicians, being the main complaints related to esthetic issues. When clinicians and patients’ evaluations were compared, it was observed that the frequencies of satisfactory restoration by patients and clinicians were similar regarding esthetic properties, and significantly different regarding functional and biological properties.

## Resumo

O objetivo deste estudo foi observar a satisfação dos pacientes com suas restaurações dentárias anteriores diretas e compará-las com a avaliação clínica do dentista usando os critérios FDI (Federation Dental International). Os pacientes pontuaram suas restaurações dentárias (n=106) anteriores em relação à satisfação (satisfatória / insatisfatória). Quando insatisfatória, ele foi entrevistado sobre a queixa. Na mesma sessão, as restaurações dentárias foram avaliadas clinicamente por dois dentistas utilizando os critérios FDI (escore 1-5) quanto aos aspectos estéticos, funcionais e biológicos. Estatística descritiva foi usada para frequências de escores atribuídos por pacientes e clínicos. Para comparar as frequências dos pacientes e dos clínicos, foi aplicado o teste Qui-quadrado (p ≤ 0,05). Os pacientes relataram suas restaurações como 52,8% satisfatórias e 47,8% insatisfatórias. Os clínicos reportaram as mesmas restaurações, 82,3% satisfatória e 17,6% insatisfatória. As queixas mais frequentes dos pacientes referiam-se à cor, seguida da forma anatômica, fratura e retenção do material e forma anatômica proximal. Comparando os índices de satisfação e insatisfação dos pacientes com os clínicos, não houve diferença em relação à estética. A frequência de restaurações insatisfatórias por dentistas foi significativamente menor quando as propriedades funcionais e biológicas foram comparadas com as opiniões dos pacientes. As restaurações foram mais frequentemente relatadas como satisfatórias pelos pacientes, sendo as principais queixas relacionadas a questões estéticas. Quando as avaliações dos clínicos e dos pacientes foram comparadas, observou-se que as frequências de restaurações satisfatórias por pacientes e clínicos foram semelhantes em relação às propriedades estéticas e significativamente diferentes em relação às propriedades funcionais e biológicas.

## Introduction

When evaluating direct anterior dental restorations, patients’ opinions regarding satisfaction and dissatisfaction are worth examining, since the reasons and approaches for repairing or replacing dental restorations can be indirectly related to esthetic or functional complaints ^1^ ^,^ ^2^ ^,^ ^3^ ^,^ ^4^.

 In clinical studies in Restorative Dentistry, patient-reported outcomes are still briefly explored. The World Dental Federation (FDI) criteria ^3^ have made an effort to include patients’ opinions as a criterion, a fact that has added to their value ^2^ ^,^ ^4^. In the “patient’s view” criterion, the patient needs to score his/her dental restoration on a 1-5 scale, as does the dentist. In this regard, the score options for patients are: 1) The patient is entirely satisfied with esthetics and function; 2) The patient is satisfied; 3) Minor criticism but no adverse clinical effects (esthetic shortcomings, some lack of chewing comfort, unpleasant treatment procedure); 4) The patient has a desire for improvement regarding esthetic and/or function; and 5) Completely dissatisfied and/or adverse effects, including pain ^3^. Despite the fact that the criterion has been proposed, Box 1 ^5^ ^-^ ^62^ shows that in 58 studies that used FDI criteria, just 17 used patient view with brief details about its approach. 

**Box 1:**
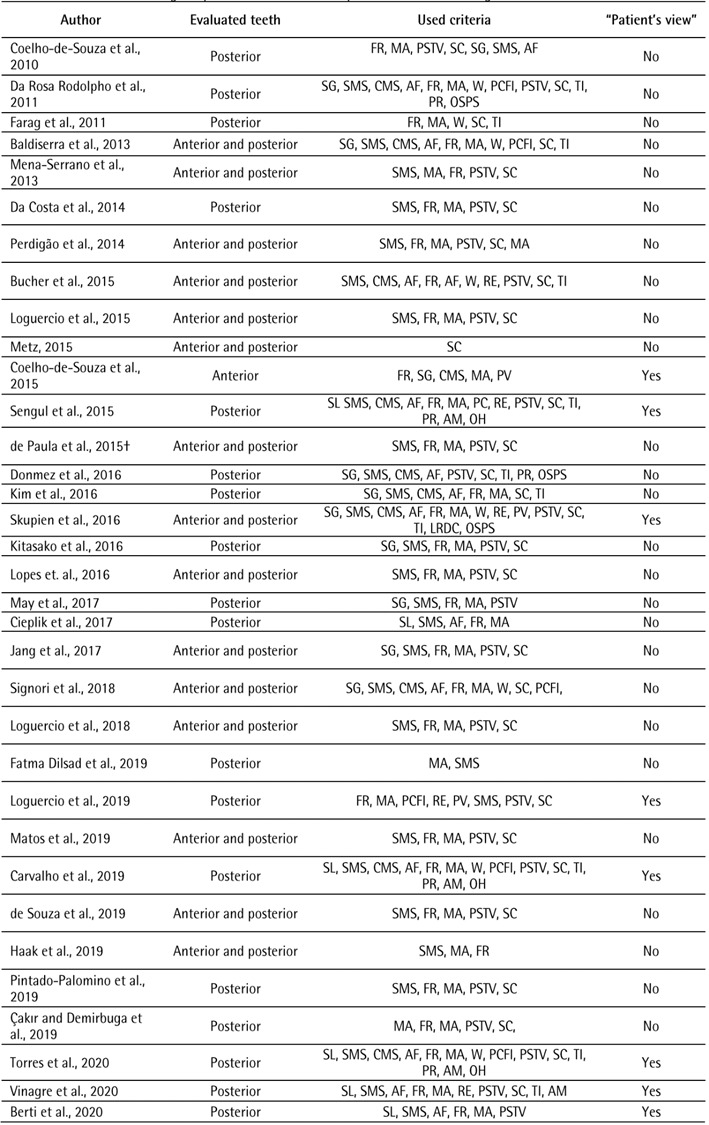
Clinical studies assessing the performance of resin composite restorations through FDI criteria.

**Box 1:**
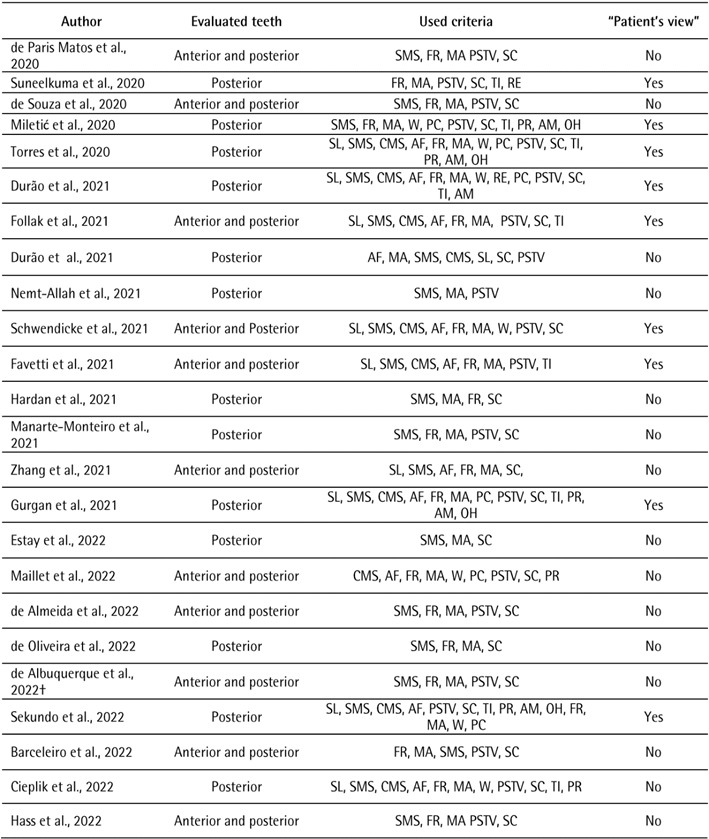
Continuation

The search strategy used the combination of keywords (FDI criteria AND composite resin AND dental restoration) in the MEDLINE/PubMed database from January 2010 to October 2022. Surface luster: SL; Surface and marginal staining: SMS; Color match/stability and translucency: CMS; Anatomical form: AF; Fracture of restorative material and retention: FR; Marginal adaptation: MA; Occlusal contour and wear: W; Proximal contact point: PC; Radiographic examination: RE; Postoperative sensitivity and tooth vitality: PSTV; Secondary caries: SC; Tooth cracks and fractures, tooth integrity: TI; Periodontal response: PR; Adjacent mucosa: AM; Oral and general health: OH.

Although the patient’s report is possibly a subjective criterion when evaluating a dental restoration ^3^, ignoring its relevance in the clinical evaluation of esthetic restorations does not help clinicians when going through the clinical decision-making process. Knowledge of the patient’s perceptions and values can be relevant in treatment decision-making, especially considering patient satisfaction ^1^ ^,^ ^2^ ^,^ ^3^ ^,^ ^4^. Additionally, it is important to understand possible discrepancies among clinician’s decision-making based on biological, functional, and esthetic criteria and patient’s demands, especially considering its implications in the repetitive restorative circle. Literature has shown that clinicians and laypersons from different locations around the world can differ in evaluating resin composite restorations ^63^ and in this context, considering a hypothetical local culture where dental esthetics were not so required we could infer that the number of interventions on dental restorations (repair and replacement) would be lower if dental restorations were functionally and biologically adequate. Such knowledge would contribute to designing national public policies and education trying to avoid the repetitive restorative circle due to minimal esthetic reasons. 

Thus, the aim of this study was to observe patients’ satisfaction regarding their direct anterior dental restorations and compare it with clinical evaluation using FDI (Federation Dental International) criteria on biological, mechanical, and esthetical domains. The null hypothesis was that the frequencies of satisfactory and dissatisfactory anterior resin composite restorations would not differ when comparing patients’ opinions with professionals’ evaluations.

### Materials and Methods

This study was approved and conducted in accordance with the local Ethic Committee (CAAE number: 34682020.5.0000.5419). The selected participants received verbal and written information concerning the study and signed the consent form. 

### Study design and sample size

This was an observational, clinical, comparative study. The anterior teeth with direct resin composite restoration were the sample unit ^15^ ^,^ ^45^ ^,^ ^48^ ^,^ ^49^. The binary outcome was the patients’ view (occurrence or nonoccurrence of satisfaction, interpreted as “satisfied” or “dissatisfied”) about their in-service anterior dental restorations. The comparison group was the professionals’ clinical evaluations of the same restorations, according to esthetical, functional, and biological domains according to FDI criteria ^3^. The sample size was calculated for an equivalence trial based on data from a pilot study (30 anterior teeth with dental restorations) where the percentage of satisfaction in the comparison group (dentist) was 65% and the percentage of satisfaction in the experimental group (patient) was 35%. Confidence was defined at 95% and power at 80%. The sample size was set at a minimum of 88 anterior teeth with dental restorations. The sequence of collecting patients’ reports and clinical evaluations was done randomly through an Excel sheet; thus, clinicians afterward they were interviewed about the same evaluated restorations, and for some other patients it was contrariwise firstly examined some patients. 

### Selection of anterior teeth with a dental restoration

This process started in February 2019 and ended in December 2019. Every patient in the first appointment in the Restorative Service at the School of Dentistry was approached. The inclusion criteria were adult patients (18-65 years old), with good general health, presenting anterior teeth with direct resin composite restorations at the buccal surface (mesial, distal, incisal, or cervical; connected or not) in upper and/or lower jaw which had been in service for at least 6 months. More than one anterior tooth with dental restoration per patient could be included since it was in the opposite dental arch and/or non-adjacent teeth. If the patient had the six anterior teeth restored, the selection considered the tooth evidence on the smile, following the sequence: ^11^ ^,^ ^21^ ^,^ ^12^ ^,^ ^22^ ^,^ ^13^ ^,^ ^23^ ^,^ ^31^ ^,^ ^41^ ^,^ ^42^ ^,^ ^32^ ^,^ ^33^ ^,^ ^43^. The exclusion criteria were endodontic-treated teeth (because tooth sensitivity was under evaluation), anterior teeth with more than one restoration, pregnant and orthodontic patients, and individuals with disabilities that make them incapable of giving an opinion about their anterior teeth with direct resin composite restorations ^15^ ^,^ ^45^ ^,^ ^48^ ^,^ ^49^. 

### Patient evaluation

The patients’ evaluations were done based on the “patient’s view” criterion, according to Hickel et al. ^3^. In the pilot study we consider the five options of scores: 1) The patient is entirely satisfied with esthetics and function; 2) The patient is satisfied; 3) Minor criticism but no adverse clinical effects; 4) The patient has a desire for improvement regarding esthetic and/or function; and 5) Completely dissatisfied and/or adverse effects, including pain. Nevertheless, scoring a restoration with five different options appeared confusing for most of our patients and some of them requested to give their opinion in terms of being satisfied/dissatisfied. Considering the FDI criteria which states that “*A simplified evaluation may be appropriate for a variety of reasons resulting in combined scores”* ^3^ we opted for combined scores 1, 2, 3 as “satisfied” (no patient desire for improvement) and 4 and 5 as “unsatisfied” (patient desiring for improvement). Thus, each patient was seated with the dental chair in the 90º position in front of a window that provided natural morning illumination (9,10,11 am) and received a facial mirror (25×18 cm with no amplification and/or light). The patient was informed where the dental restoration to be evaluated was positioned and it was asked: “Is this dental restoration satisfactory for you? If not, what bothers you about it?”. The answers were recorded in terms of a) “Entirely satisfied with esthetics and function”, meaning that no procedure was involved, or b) “Dissatisfied”, meaning that repair or replacement could be involved ^3^. The reasons for dissatisfactory anterior resin composite restorations were recorded using the patient’s words. The complaints were summarized and classified as being esthetical (color, stain, shape, size) or functional (roughness and crack) as, according to Hickel et al. ^3^, the patient can only report the reason for a dissatisfactory restoration as being esthetical or functional.

### Clinical evaluation

In the same session, two clinicians clinically evaluated the dental restorations, independently. The professionals were experienced (more than 10 years in the field of restorative dentistry - AESG and SAMC), and calibrated through a local portfolio of digital dental restoration images. A total agreement score of ≥85% ^2^ ^)^ was obtained. The evaluations of the selected dental restorations on anterior teeth were made using the following: esthetic criteria (surface luster; surface staining; color match/translucency; esthetic anatomical form); functional criteria (fractures and retention; marginal adaptation; wear and occlusal contour; approximal form and contact point) and biological criteria (tooth sensitivity and vitality; recurrence of carious lesion, erosion, abfraction; tooth integrity; periodontal response; adjacent mucosa; oral health). The scores ranged from 1 (clinically excellent/very good); to 2 (clinically good); 3 (clinically sufficient/satisfactory); 4 (clinically unsatisfactory but repairable) and 5 (clinically poor/replacement necessary). A researcher (BNF) who was not involved in the assessment of the dental restorations recorded the responses.

### Data analysis

The absolute and relative frequencies of scores attributed by patients and dentists to anterior teeth with direct resin composite restoration were observed using descriptive statistics. In order to analyze the data, clinical scores were grouped considering 1, 2, and 3 as satisfactory, 4 (repair), and 5 (replacement) as dissatisfactory ^3^. The Chi-square test was used to compare the frequencies. In all tests, the level of significance was set at p ≤ 0.05, and calculations were performed using the IBM statistics version 20.0 for Windows (IBM Corp., Armonk, Nova York, United States).

## Results

Initially, 124 patients were assessed; from that 56 patients were included according to inclusion criteria (21 male, 35 female) being a mean of 55 years old (40-77). A total of 106 anterior teeth with resin composite restorations (one restoration per tooth; mean of 1.8 anterior teeth per patient) were evaluated by patients and clinicians: 16 on upper right canines (#13), 13 on upper right lateral incisor (#12), 17 on upper right central incisor (#11), 12 on upper left central incisor (#21), 15 on upper left lateral incisor (#22), 11 on upper left canine (#23), seven on lower left canine (#33), five on lower left lateral (#32), one on lower left central ^31^, three on lower right central incisor (#41), one on lower right lateral incisor (#42), and five on lower right canine (#43), tooth # are according to the international nomenclature.

**Figure 1:**
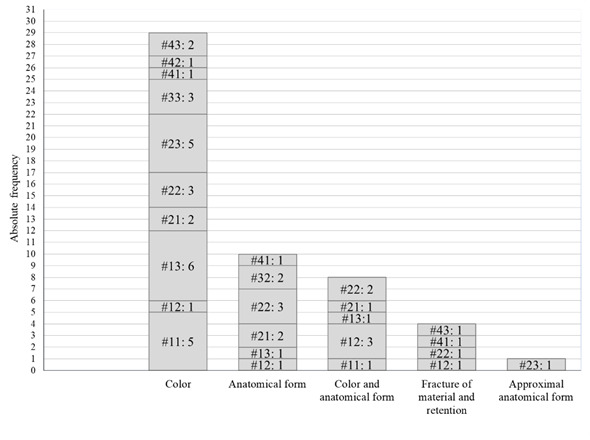
Graphic illustration of absolute frequency regarding the reasons for dissatisfaction in patients’ reports on their anterior resin composite restorations. Data (column) is organized by reasons. The column represents the total absolute frequency and is divided according to the absolute frequency of each tooth, which is identified by #teeth number followed by: corresponding absolute frequency.

Patients reported 52.8% of their in-service anterior resin composite restorations as satisfactory and 47.8% as dissatisfactory. Not all patients were able to disclaim the reasons for dissatisfactory anterior resin composite restoration. Figure 1 shows the reason for dissatisfaction where the most frequent complaint was color (55,7%), followed by anatomical form (19,2%), color and anatomical form (15,3%) fracture of the material and retention (7,6%), and approximal anatomical form (1.9%). Interestingly, the overall rate for clinician’s satisfaction or dissatisfaction with the same direct anterior resin composite restorations were 82,3% and 17,6% respectively. The outcomes from the comparison between clinicians and patients are shown in Table 1. Comparing patients’ reports of satisfaction or dissatisfaction with each FDI criterion evaluated by clinicians, statistical difference was found in fracture of material and retention (p = 0.007), wear and occlusal contour (0.001), approximal anatomical form, and contact point (p = 0.011), sensitivity and tooth vitality (p < 0.001), recurrence of caries, erosion, abfraction (p < 0.001), tooth integrity (p < 0.001), periodontal response (p < 0.001), adjacent mucosa (p < 0.001), and oral health (p < 0.001). Statistical differences were not seen when patients’ reports were compared with the dentists’ outcomes in aesthetics Summarizing, for the esthetic criteria, the percentages of satisfactory and dissatisfactory anterior resin composite restorations were similar between dentists and patients. For functional and biological properties, the frequency of dissatisfaction given by clinicians decreased, becoming statistically different from patients’ reports. 

**Table 1:** Absolute and relative frequency of scores for the clinically assessed criteria.

FDI criteria	1. Surface luster		2. Surface staining		3. Color match, translucency		4. Anatomical form		5. Fracture of material and retention		6. Marginal adaptation		7. Wear and occlusal contour		10.Patient’s view	
	N	%	N	%	N	%	N	%	N	%	N	%	N	%	N	%
Satisfactory†	58	54.7	64	60.4	54	50.9	60	56.6	75	70.8	69	65.1	79	74.5	56	52.8
Unsatisfactory‡	48	45.3	42	39.6	52	49.1	46	43.4	31	29.2	37	34.9	27	25.5	50	47.2
P-value*	0.783		0.268		0.783		0.581		0.007*		0.070		0.001*		Reference	
	8. Approximal anatomical form, and contact point		11. Sensitivity and tooth vitality		12. Recurrence (caries, erosion, abfraction)		13. Tooth integrity		14. Periodontal response		15. Adjacent mucosa		16. Oral health		Dentist´s overall rate	
	N	%	N	%	N	%	N	%	N	%	N	%	N	%	%	
Satisfactory†	74	69.8	106	100	105	99.1	105	99.1	105	99.1	106	100	106	100	82.35	
Unsatisfactory‡	32	30.2	0	0.0	1	0,9	1	0,9	1	0,9	0	0.0	0	0.0	17.65	
P-value*	0.011		<0.001*		<0.001*		<0.001*		<0.001*		<0.001*		<0.001*			

†Satisfactory refers to scores 1, 2 and 3; ‡ Unsatisfactory refers to scores 4 and 5 on FDI criteria. *P-Value statistically significant (p ≤ 0.05) refers to the comparison between patient´s view FDI criterion reference value ^10^ and each esthetic ^1^ ^,^ ^2^ ^,^ ^3^ ^,^ ^4^, functional ^5^ ^,^ ^6^ ^,^ ^7^ ^,^ ^8^ and biological FDI criteria ^11^ ^,^ ^12^ ^,^ ^13^ ^,^ ^14^ ^,^ ^15^ ^,^ ^16^, coming from dentists assessment.

## Discussion

In this study, patients viewed their teeth with direct anterior resin composite restorations as 47.8% non-satisfactory, thus demanding intervention and the reasons were mainly color and anatomical form (80%). Besides, it was found that clinicians were overall mostly satisfied (82.3%) with these same anterior resin composite restorations, mainly regarding their functional and biological aspects. When clinicians' evaluations were observed separately it is possible to observe that clinicians and patients rates for esthetics are similar. These findings are important because they suggest a trend for the repair and replacement of resin composite restorations based on esthetic demand, despite its proper functional and biological aspects. 

Concerning the methodology, patients in this study were at the first appointment of the Restorative Service and the reasons for being scheduled were various (seeking for dental bleaching; dissatisfaction with posterior or anterior restorations, and others) as the checking-in approach in the service is by free-demand. The patients’ opinions were collected by a third researcher who was not involved in the clinical assessments. Randomization was applied to guarantee that half of the patients had been clinically examined before giving their opinions; the other half gave their opinions after being examined because the time spent in the assessment of FDI criteria by two professionals (which is long considering all the criteria to be evaluated) could exhaust the patient, leading to possible bias when their opinions were requested. Also, patient's opinions were collected per tooth; meaning that the same patient could opine for more than one restored tooth in his/her oral cavity. In this sense, this study had a 1.8 restored tooth included per patient, which is in accordance with the literature ^15^ ^,^ ^45^ ^,^ ^48^ ^,^ ^49^. The study had a pilot test with a methodological approach based on FDI criteria responses. Initially, all scores were considered in a pilot study, however, our sample showed difficulty and uncertainty in providing enough information for categorization and discrimination between scores with minor differences. Then, the threshold established for statistical analysis was dichotomized into patient’s satisfaction (no intervention demanded) or dissatisfaction (intervention demanded). Consequently, in terms of data analysis, clinicians’ scores 1, 2, and 3 (maintain) were allocated as satisfactory and 4 (repair) and 5 (replacement) were allocated as dissatisfactory, which helped the investigators to analyze patients’ reports and the clinical decision-making process. Hickel et al. ^3^ mention this scheme of grouped scores as appropriate. Indeed, it is suggested the definition of criteria analysis be used before the starting of clinical evaluation according to the intended purpose, as was performed in the pilot study ^3^. 

For the clinical evaluation, both professionals made their evaluations blinded to patients’ reports of satisfaction or dissatisfaction to avoid influence in clinical decision-making. Patients were aware that clinicians would be evaluating their anterior dental restorations at the moment they signed the Consent Form; nevertheless, the result of the clinical decision-making process was not disclosed, and researchers (AESG and SMC) communicated to each other using the numbers attributed to criteria, as in Table 1, and the FDI scores; thus, it is possible to assume that the patients were not aware of the clinicians’ evaluations. The clinical studies that evaluated patient satisfaction through the “patient’s view” criterion, briefly described how the assessment was performed possibly because the purpose of those clinical trials was to evaluate experimental materials and techniques, mostly on posterior teeth, and also because they used different criteria, other than patients’ satisfaction as evidence ^5^ ^,^ ^20^ ^,^ ^29^ ^,^ ^31^ ^,^ ^36^. Furthermore, it is worth mentioning that patients’ satisfaction in such studies ranged from 90 to 100%, which contrasts with the rate found in this study. 

Discussing the results of this study, patients’ reports comprised 52.8% of satisfactory and 47.8% of dissatisfactory anterior resin composite restorations. As the “patient’s view” criterion includes an interview, the researchers in this study organized patients’ complaints as shown in Figure 1. Overall, the reported causes for patient dissatisfaction were mainly color (55.7%), followed by anatomical form (19.2%), color and anatomical form (15.3%) fracture of the material and retention (7.6%), and approximal anatomical form (1.9%). Interestingly, the esthetical complaints meant 90% of the reasons for dissatisfaction of patients and there were no complaints related to biological properties (e.g., tooth sensitivity, gingival bleeding). The rate of dissatisfaction reported by the patients in this study contrasts with investigations that applied the “patient’s view” criterion showing greater rates (95.8-100%) for satisfactory posterior ^29^ ^,^ ^31^ ^,^ ^36^ ^,^ ^37^ ^)^ posterior and anterior ^20^ and anterior ^15^ resin composite restorations. One point to consider when discussing this contrast is the difference in the methodology since they are clinical trials that evaluate restorations made with a certain material and under controlled conditions ^15^ ^,^ ^20^ ^,^ ^29^ ^,^ ^31^ ^,^ ^36^ ^,^ ^37^. Although Coelho and Souza et al. ^15^ evaluated 142 anterior resin composite restorations and all patients considered the restorations satisfactory, the restorations were performed by the same group of operators (postgraduate students during Operative Dentistry courses) in a controlled environment. In this study, the restorations were performed by unknown different professionals, using various types of materials and possibly techniques, and were in service for a minimum of six months; such heterogeneity can lead to a lower level of satisfaction (considering both patients and dentists) when compared with data from clinical trials where the operating conditions can be ideal. Corroborating this assumption, a recently published practice-based report showed that the need for re-intervention in dental restoration was about 70% ^4^. Regarding patients’ causes for dissatisfaction, in this study, the most expressive rates of dissatisfaction were related to upper teeth (left and right) canines, and were due to “color”, as can be observed in Figure 1. In this sense, a critical review from Demarco et al. ^2^ showed that the factors which affect the longevity in anterior and posterior teeth are different; being esthetic demands (color mismatch and surface or marginal staining) the predominant reason for intervention on anterior teeth. Considering the data of this study, upper teeth were majorly included (89 upper vs 25 lower), which can explain the rates of dissatisfaction related to this dental group. In addition, the reasons for dissatisfaction with upper canines can be justified by the fact of canines play an important role in frontal dentofacial esthetics ^1^ ^,^ ^2^ ^,^ ^4^, and also by the fact that canines are naturally more dark/red/yellow than the other anterior teeth, which may have interfered with patients’ understanding ^1^ ^,^ ^2^ ^,^ ^4^. 

Detailing the clinical evaluation, 14 FDI criteria were assessed in this study. The frequencies of satisfactory anterior dental restorations by clinicians were also lower than the ones reported in clinical trials involving anterior dental restorations regarding esthetic properties. For instance, the surface luster was found satisfactory in 54.7% of cases, contrasting with Skupien et al. ^20^ who found 95.8% of satisfaction; for staining, the present study found a 60.4% satisfaction rate, contrasting with a 100% satisfaction rate found by Coelho-de-Souza et al. ^15^. Considering color match and translucency, 50.9% of the restorations were found satisfactory, while other studies reported 100% ^15^ ^)^ or 95.8% ^20^ ^)^ satisfaction rates. For esthetic anatomical form, 56.6% of the restorations were found satisfactory, also contrasting with the 100% satisfaction rates reported in those studies ^15^ ^,^ ^20^. In relation to functional properties, professionals’ satisfaction regarding fracture of material and retention (70.8%), marginal adaptation (65.1%) wear and occlusal contour, (74.5%), approximal anatomical form and contact point (69.8%) was again lower than in other studies where the satisfaction rates varied from 91.3 to 100% ^15^ ^,^ ^20^. It is interesting to note that in this study both patients and clinicians were similarly less satisfied than their pairs in controlled clinical trials concerning anterior dental restorations, especially regarding esthetic properties. Such a situation can be explained by the heterogeneity of the sample, with various types and brands of resin composite material, time in service, and professionals’ ability, among others. In this sense, it appears reasonable that the satisfaction or success rate of dental restorations can be greater in clinical trials. Contrastingly, in this study, biological properties received expressive rates of satisfaction from dentists. For example, sensitivity and tooth vitality (100%), recurrence of caries (99.1%), tooth integrity (99.1%), periodontal response and adjacent mucosa (99.1%), oral and general health (100%). The expressive percentage of satisfactory biological properties in the anterior resin composite restorations was not expected, as esthetic and functional properties performed poorly compared with the available literature. Nevertheless, this indicates that resin composite restorations are being performed to preserve dental biology and oral health, and/or that patients were mostly committed to dental hygiene procedures.

Considering patients and dentists, it is worth mentioning that the main reported cause for dissatisfaction among patients was “color” while among dentists it was surface luster (54.7%), staining (60.4%), color match, and translucence (50.9%). From this panorama, one can extrapolate that issues related with surface luster, staining, color matching, and translucence might be interpreted by patients as “color”, and consequently, patients’ needs for improvement were similarly perceived by the dentists. With dentists and patients showing a similar trend parameters, which bring advantages, such as a broader range of information, and disadvantages, such as possible difficulties regarding the comparison with other studies ^1^ ^,^ ^2^ ^,^ ^3^ ^,^ ^4^.

Among the limitations of this study are the absence of similar studies to compare and discuss data regarding patients’ reports in Restorative Dentistry. In this sense, practice-based studies would include a patient-centered approach. Additionally, demographic data on patients could assist in understanding how it supposedly influences their opinions. 

Therefore, according to the objectives investigated, the following conclusions were found: patients’ views about their in-service direct anterior dental restorations were 52.8% satisfactory and 47.8% not satisfactory. Overall, clinicians reported the same restorations as 82,3% satisfactory and 17.6% not satisfactory. The patients’ dissatisfaction was mainly related to color, anatomical form, fracture of material and retention, and approximal anatomical form. When clinical evaluation per domain and patient evaluations were compared, it was seen that the frequencies of satisfactory restoration by patient and dentist were similar for esthetic properties and significantly different for functional and biological properties.
